# Quorum Sensing-Dependent Invasion of Ralstonia solanacearum into Fusarium oxysporum Chlamydospores

**DOI:** 10.1128/spectrum.00036-23

**Published:** 2023-06-27

**Authors:** Chiaki Tsumori, Shoma Matsuo, Yuta Murai, Kenji Kai

**Affiliations:** a Graduate School of Agriculture, Osaka Metropolitan University, Osaka, Japan; Connecticut Agricultural Experiment Station

**Keywords:** *Fusarium oxysporum*, *Ralstonia solanacearum*, endoparasitism, quorum sensing

## Abstract

Strains of the Ralstonia solanacearum species complex (RSSC), although known as the causative agent of bacterial wilt disease in plants, induce the chlamydospores of many fungal species and invade them through the spores. The lipopeptide ralstonins are the chlamydospore inducers produced by RSSC and are essential for this invasion. However, no mechanistic investigation of this interaction has been conducted. In this study, we report that quorum sensing (QS), which is a bacterial cell-cell communication, is important for RSSC to invade the fungus Fusarium oxysporum (*Fo*). Δ*phcB*, a deletion mutant of QS signal synthase, lost the ability to both produce ralstonins and invade *Fo* chlamydospores. The QS signal methyl 3-hydroxymyristate rescued these disabilities. In contrast, exogenous ralstonin A, while inducing *Fo* chlamydospores, failed to rescue the invasive ability. Gene-deletion and -complementation experiments revealed that the QS-dependent production of extracellular polysaccharide I (EPS I) is essential for this invasion. The RSSC cells adhered to *Fo* hyphae and formed biofilms there before inducing chlamydospores. This biofilm formation was not observed in the EPS I- or ralstonin-deficient mutant. Microscopic analysis showed that RSSC infection resulted in the death of *Fo* chlamydospores. Altogether, we report that the RSSC QS system is important for this lethal endoparasitism. Among the factors regulated by the QS system, ralstonins, EPS I, and biofilm are important parasitic factors.

**IMPORTANCE** Ralstonia solanacearum species complex (RSSC) strains infect both plants and fungi. The *phc* quorum-sensing (QS) system of RSSC is important for parasitism on plants, because it allows them to invade and proliferate within the hosts by causing appropriate activation of the system at each infection step. In this study, we confirm that ralstonin A is important not only for Fusarium oxysporum (*Fo*) chlamydospore induction but also for RSSC biofilm formation on *Fo* hyphae. Extracellular polysaccharide I (EPS I) is also essential for biofilm formation, while the *phc* QS system controls these factors in terms of production. The present results advocate a new QS-dependent mechanism for the process by which a bacterium invades a fungus.

## INTRODUCTION

The interactions between bacteria and fungi play a vital role in natural and agricultural ecosystems (1–3). They are cornerstone members of communities riding biochemical cycles in plant rhizosphere and contribute to plants’ health and diseases (4–6). However, our knowledge of interactions among microbes is less than that of interactions between plants and microbes. The most intimate examples of microbial interactions are bacterial parasitism/symbiosis in fungal hosts (3, 7, 8). Recent advances have shown that some bacteria can live inside fungal cells (e.g., hyphae and asexual spores) by forming endosymbiotic relationships with their host fungi. These bacteria, called endobacteria or endofungal bacteria, have now gained much attention in the studies of microbial ecology and the evolution of endocellular symbiosis. Unfortunately, there are many examples of endobacteria that cannot grow outside the fungal cells. Even when cultured, reinfection experiments have been accomplished in a limited number of cases (3, 7, 8).

The association of Mycetohabitans rhizoxinica (formerly Burkholderia rhizoxinica) with Rhizopus fungi is a well studied example of endobacterium-fungus interactions (9). Rhizopus microsporus, a mucoromycete fungus, is the causative agent of rice seedling blight. It produces the phytotoxin rhizoxins responsible for the characteristic symptoms (10, 11). Partida-Martinez, Hertweck, and colleagues showed that the toxins are produced by the combination of M. rhizoxinica and R. microsporus (12, 13). The bacterial cells invade the fungal hyphae by degrading the cell walls with chitinase and may escape fungal defense using an unusual galactofuranose lipopolysaccharide (14, 15). The endobacterium-free R. microsporus cannot produce rhizoxins and cannot form asexual spores (12, 16). Later, the bacterial endosymbiont was found also to influence the sexual reproduction of the fungus host (17). Generally, the endobacterium is essential for the host lifestyle.

**FIG 1:**
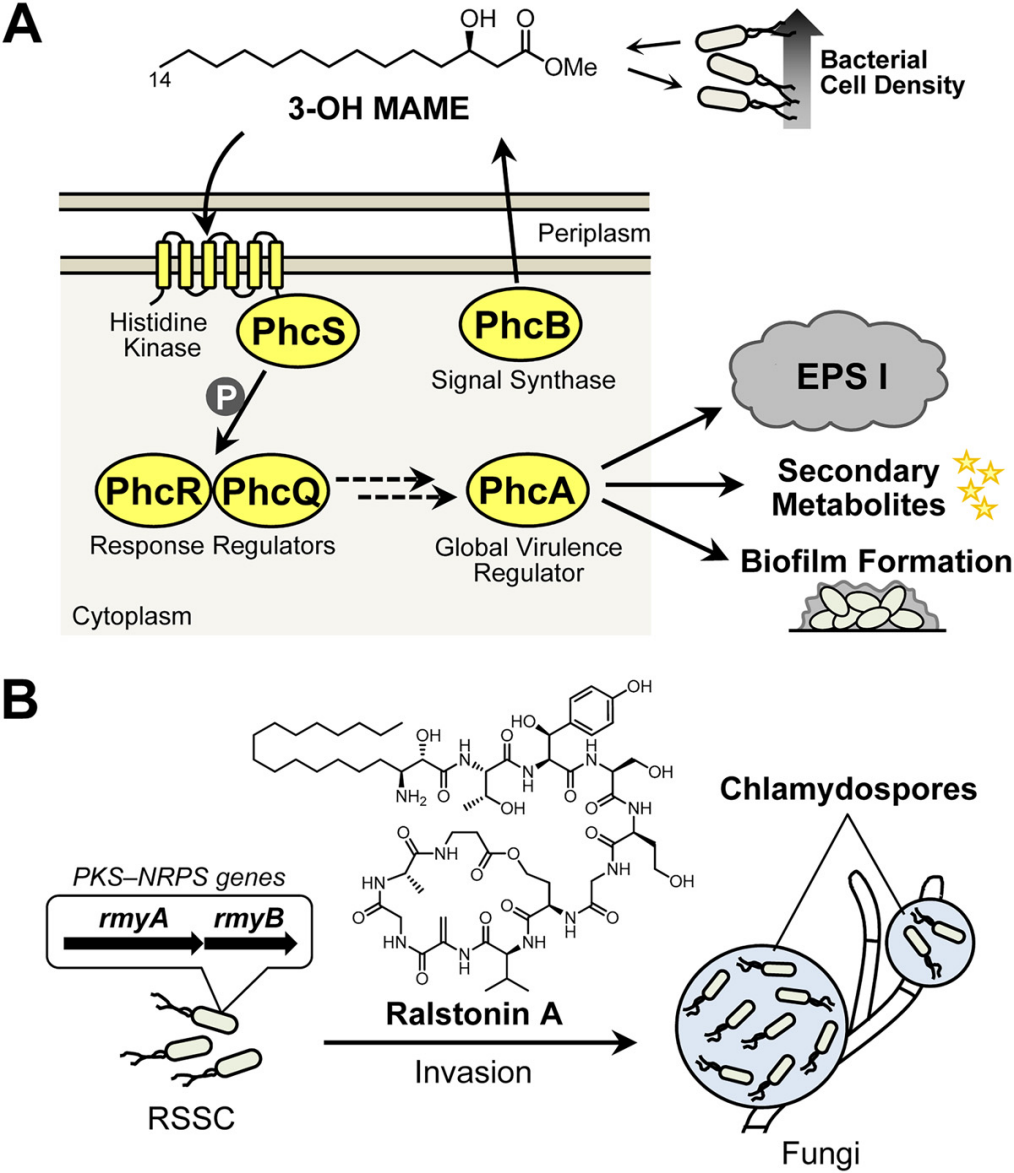
*phc* quorum-sensing (QS) system of the Ralstonia solanacearum species complex (RSSC) and its endoparasitism in fungi. (A) Schematic diagram of the *phc* QS system in strain OE1-1. As bacterial density increases, (*R*)-methyl 3-hydroxymyristate (3-OH MAME) concentrations rise, and the molecule is received by PhcS-PhcRQ. Then, PhcA is functionalized and induces the production of virulence factors, such as extracellular polysaccharide I (EPS I), secondary metabolites, and biofilms. (B) Possible endoparasitism of RSSC coupled with chlamydospore induction. Ralstonin A synthesized by the PKS-NRPS induces chlamydospore formation in many fungi and may facilitate endoparasitism.

Strains of Ralstonia solanacearum species complex (RSSC) are known as the causative agents of bacterial wilt disease on solanaceous plants (18, 19). Studies on RSSC-host plant interactions have been accumulated. Therefore, we can report, in detail, how the bacteria invade host plant tissues and cause wilting symptoms (19, 20). Quorum sensing (QS), a bacterial cell-cell communication, plays a crucial role in RSSC virulence expression in plants, where the *phc* regulatory elements encoded by the *phcBSRQ* operon and *phcA* gene play pivotal roles (18, 19). Previously, we reported that the RSSC strain OE1-1 employs (*R*)-methyl 3-hydroxymyristate (3-OH MAME) as the *phc* QS signal (Fig. 1A) (21). The methyltransferase PhcB synthesizes 3-OH MAME, and the two-component system PhcS-PhcRQ senses the QS signal, which leads to the activation of the global virulence regulator PhcA and the subsequent *phc* QS circuit (21, 22). Deleting *phcB* or *phcA* dramatically decreased virulence in host plants (18, 22). Cell-cell chemical communication is essential for RSSC virulence in plants.

In 2016, Keller and coworkers (23) found that RSSC strain GMI1000 can induce chlamydospore formation in phylogenetically diverse fungi (including members of *Mucoromycota*, *Ascomycota*, and *Basidiomycota*) and invade the fungal cells probably through the formed chlamydospores (Fig. 1B). They also suggested that the polyketide synthase-nonribosomal peptide synthetase (PKS-NRPS) enzymes encoded by *rmyA*/*rmyB* genes may produce a pivotal compound in inducing chlamydospores. We later identified ralstonins A and B, unique cyclic lipopeptides from RSSC strains OE1-1 and GMI1000, to be the chlamydospore inducers for Fusarium oxysporum (Fig. 1B) (24). One month later, Nett and coworkers (25) also reported the chemical characterization of the ralstonins (also known as ralsolamycins). However, since we have just begun to understand the results of these RSSC-fungi interactions, little is currently known about how and why the bacteria invade fungal cells. We speculated whether some commonality existed between this endoparasitism and plant-wilt disease development; thus, is the RSSC *phc* QS system involved in endoparasitism?

A quantitative assay for evaluating the invasion rate of RSSC cells into F. oxysporum chlamydospores was developed in this study. We investigated the key factors establishing this interkingdom interaction by analyzing the invasion rates of the gene-deletion and -complementation RSSC mutants. A coculturing assay to directly observe interactions between RSSC and F. oxysporum was also established. Consequently, ralstonin/extracellular polysaccharide I (EPS I) production and biofilm formation, both of which are regulated by the *phc* QS system, were essential for endoparasitism establishment. Alternatively, extracellular enzymes and type II/III secretion systems are not involved, although these are important factors for plant infection. Insight into the behaviors of RSSC cells after the invasion of F. oxysporum chlamydospores was obtained using transmission electron microscopy (TEM) and fluorescence microscope. Based on these findings, snapshots of the course of the QS-dependent entry of RSSC cells into F. oxysporum chlamydospores was proposed.

## RESULTS

### Ralstonins induce F. oxysporum chlamydospores and RSSC invasion.

**FIG 2:**
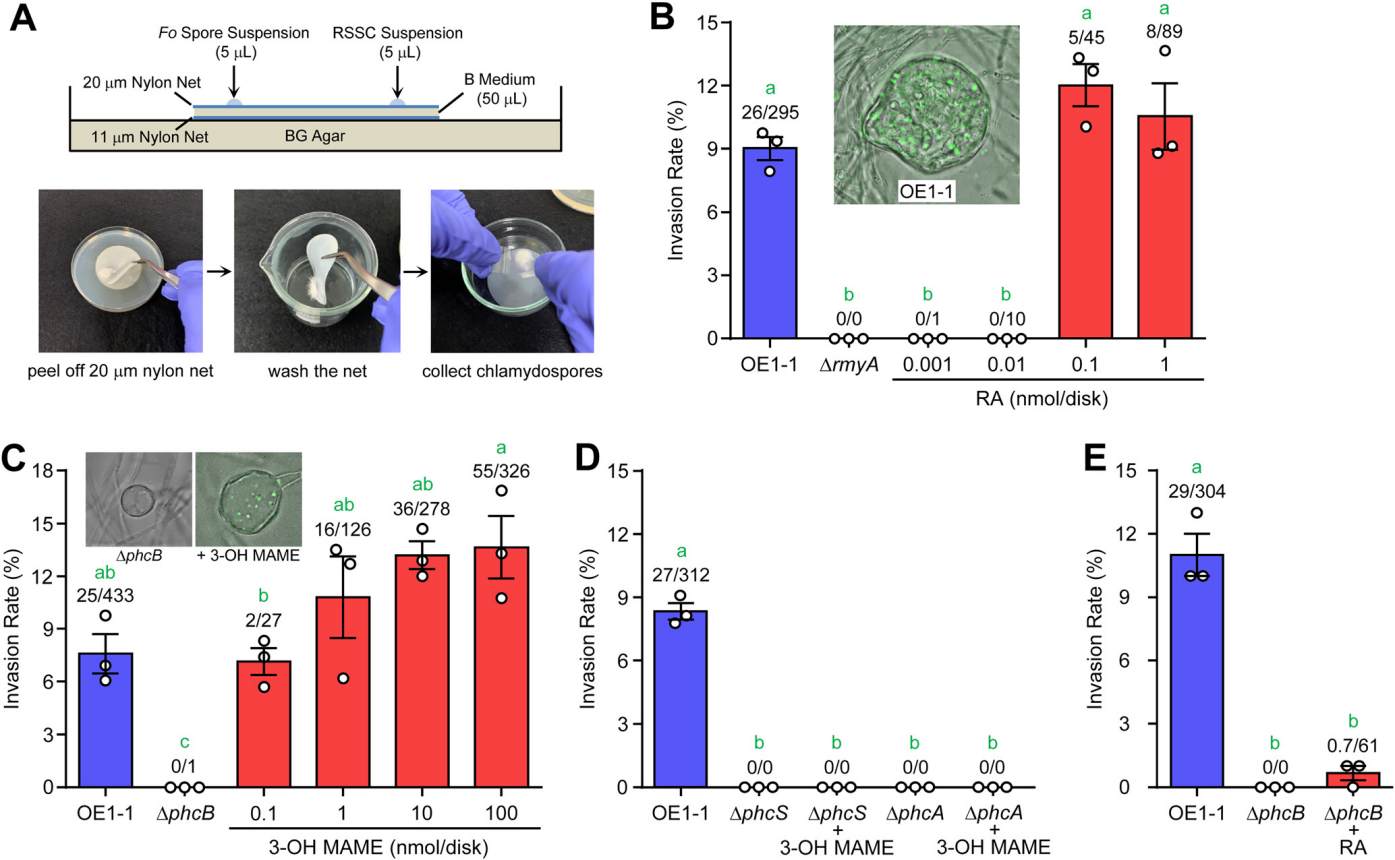
Analysis of endoparasitism of the RSSC strain OE1-1 and its mutants in F. oxysporum. (A) Nylon net sandwich assay to evaluate the invasion rate of RSSC strains. The images of assay plate (upper) and chlamydospore recovery method (lower) are shown. (B) Invasion rates of OE1-1 and Δ*rmyA* into F. oxysporum chlamydospores. The addition of ralstonin A (RA) rescued the invasion rate of Δ*rmyA*. A merged photo of a chlamydospore infected with OE1-1 is shown as the inset. (C) Evaluation of the invasion ability of Δ*phcB* into F. oxysporum chlamydospores and the effect of 3-OH MAME. The addition of 3-OH MAME rescued the invasion rate of Δ*phcB*. Merged images of a chlamydospore induced by Δ*phcB* and a chlamydospore invaded by Δ*phcB* with 3-OH MAME treatment are shown in the inset. (D) Invasion rates of Δ*phcS* and Δ*phcA* and their response to 3-OH MAME (1 nmol/disk). (E) The effect of ralstonin A (0.1 nmol/disk) on the invasion of Δ*phcB*. The error bars show the mean ± standard error of the mean (SEM; *n *= 3). The numbers above the bars indicate infected spores/total spores. The green letters above the bars show significant difference (*P < *0.05, Tukey’s test).

The RSSC strain OE1-1 aggressively induces the chlamydospore formation of F. oxysporum NBRC 31213 and invades the spores when they are cocultured (24, 26). F. oxysporum strains form fewer conidia than Aspergillus spp., making it suitable for microscopic observation. A nylon net sandwich assay that assessed the invasion rate of this bacterium into the chlamydospores of F. oxysporum using a constitutive green fluorescent protein (GFP)-producing vector (pDSK-GFP) was developed (Fig. 2A). In this method, the invasion rate can be calculated efficiently by washing the nylon net to remove excess RSSC cells and collecting the formed chlamydospores with a cover glass. Strain OE1-1 (wild-type) showed 8.8% invasion into the formed chlamydospores (Fig. 2B). The invasion rate of the wild-type strain was around 10% throughout the experiments conducted in this paper. Δ*rmyA*, a ralstonin-deficient mutant of strain OE1-1 (24, 27), did not induce the chlamydospores and thus could not establish the endoparasitism (Fig. 2B). The exogenous ralstonin A (0.1 and 1 nmol/disk) rescued the chlamydospore formation and endoparasitic invasion of Δ*rmyA*. According to these results, ralstonin-dependent chlamydospore formation is needed for RSSC’s entry into the spores, and this assay can quantitatively evaluate the invasion rates of RSSC strains.

### QS-deficient mutants lose their invasion ability into F. oxysporum chlamydospores.

The virulence of RSSC on plants is tightly regulated by the *phc* QS system (18, 22). Therefore, we wondered whether the *phc* QS system is also involved in the interaction of RSSC with F. oxysporum. To examine this, we investigated the invasion ability of Δ*phcB* (a QS signal synthase-deletion mutant) (21) into F. oxysporum. Δ*phcB* neither induced the chlamydospore formation of F. oxysporum nor established the endoparasitism on the fungus (Fig. 2C). Exogenous 3-OH MAME dose-dependently rescued the invasion rate of Δ*phcB*. When 3-OH MAME was applied (0.1 nmol/disk), Δ*phcB* exhibited similar invasion rates compared with the wild-type strain. Also, 3-OH MAME increased the invasion rate over the wild-type strain at higher concentrations. Δ*phcS* (QS signal receptor-deficient) and Δ*phcA* (the global QS regulator deficient) (21, 28) also lost the invasion ability into the chlamydospores, which was not restored by the 3-OH MAME supplement (Fig. 2D). According to these results, the *phc* QS circuit signaling activated by 3-OH MAME is important for RSSC invasion into F. oxysporum chlamydospores.

The production of ralstonins is under the control of the *phc* QS system in the OE1-1 strain (Fig. S1) (24). Furthermore, we checked whether the loss in invasion ability of Δ*phcB* and the related mutants is attributed only to the deficiency of ralstonin production. Consequently, while ralstonin A (0.1 nmol/disk) induced the formation of many chlamydospores, the compound did not rescue the invasion rate of Δ*phcB* to that of strain OE1-1 (Fig. 2E). The concentration of ralstonin A used here was one that rescued the invasion rate of Δ*rmyA* (Fig. 2B). Δ*phcS* and Δ*phcA* results were similar to those observed in the Δ*phcB* strain (Fig. S2). Therefore, it was concluded that other factor(s) regulated by the *phc* QS system are also crucial for establishing RSSC parasitism.

### Extracellular enzymes and type II/III secretion systems are not involved in RSSC invasion.

**FIG 3:**
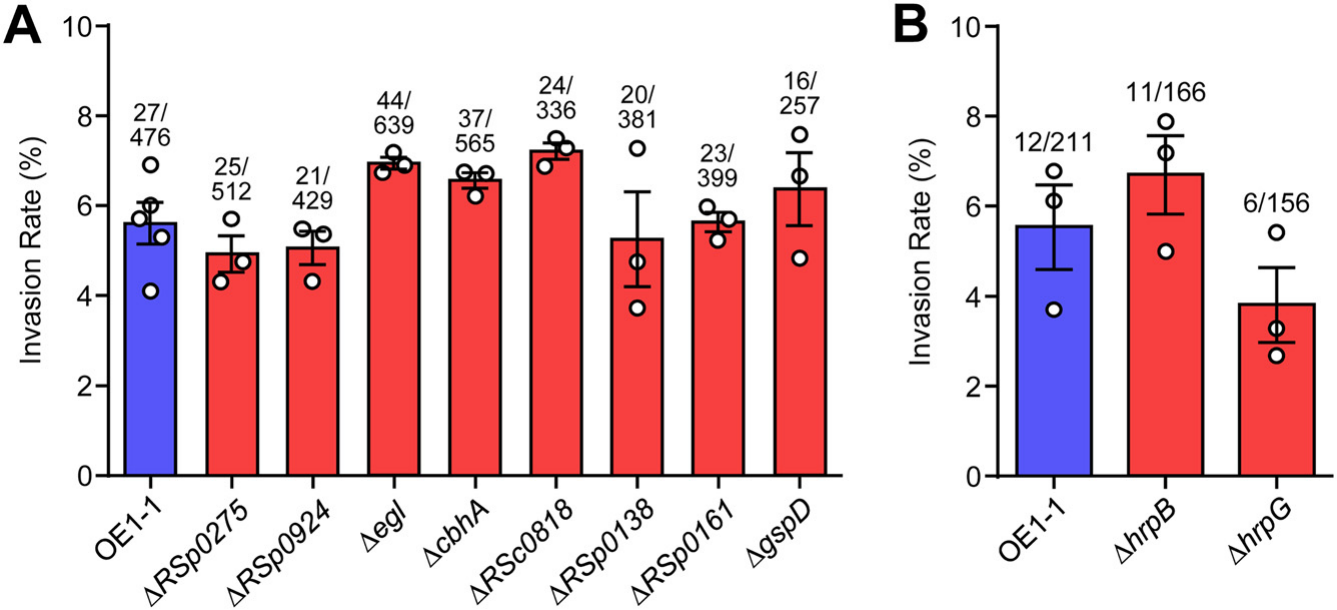
Evaluation of extracellular enzymes, type II secretion system (T2SS), and type III secretion system (T3SS) on RSSC endoparasitism in F. oxysporum. (A) Invasion rates of the gene-deletion mutants of extracellular enzymes and the T2SS component into F. oxysporum chlamydospores. Chitinase genes (*RSp0275* and *RSp0924*), glucanase genes (*cbhA*, *egl*, and *RSc0818*), lipase genes (*RSp0138* and *RSp0161*), and a T2SS machinery gene (*gspD*) were used. (B) Invasion rates of Δ*hrpB* and Δ*hrpG* into F. oxysporum chlamydospores. The error bars show the mean ± SEM (*n *= 3 or 5). The numbers above the bars show infected spores/total spores. No statistically significant differences in data set values were recorded against the control (*P* values for both strains were >0.05, Dennett’s test).

To invade F. oxysporum chlamydospores, the RSSC cells should overcome their physical barriers (e.g., cell membranes and walls) (15). As expected, this bacterium harbors two chitinase genes (*RSp0275* and *RSp0924*), three glucanase genes (*cbhA*, *egl*, and *RSc0818*), and two lipase (or esterase) genes (*RSp0138* and *RSp0161*) (29). The previous RNA sequencing (RNA-Seq) comparison between OE1-1 and Δ*phcB* showed that the expression of these genes is under the control of the *phc* QS system (30–32). We prepared gene-deletion mutants and evaluated their invasion ability into F. oxysporum chlamydospores. However, these deletion mutants invaded chlamydospores at similar rates to the wild-type strain (Fig. 3A). Because many extracellular enzymes (including uncharacterized ones) are secreted by type II secretion system (T2SS) (19), we created its deficient mutant (Δ*gspD*; *gspD* encodes an important protein of T2S machinery) (33) and conducted the invasion assay. Δ*gspD* also showed a similar invasion rate as strain OE1-1 (Fig. 3A). Fungal cell membranes and walls may not be a barrier for RSSC to accomplish this invasion, or they may be using a completely different method. We concluded that such extracellular enzymes are not critical parasitic factors regulated by the *phc* QS system.

Previously, the importance of type III secretion system (T3SS) in M. rhizoxinica-R. microsporus interaction was described (34). T3SS is a specialized syringe-shaped protein-export system used by pathogenic Gram-negative bacteria to inject virulence proteins (effectors) into host cells, and in many cases, it is important for bacterial pathogenicity (35, 36). In RSSC, the expression of T3SS components is regulated by the *phc* QS system and is upregulated or downregulated depending on the culturing conditions (37, 38). We examined the invasion rates of Δ*hrpB* and Δ*hrpG* to check the importance of T3SS in RSSC-F. oxysporum interaction. HrpB and HrpG are positive RSSC T3SS regulators, with *hrpB* gene transcription activated by HrpG (39, 40). Δ*hrpB* showed no significant decreases in invasion rates into F. oxysporum chlamydospores compared with the wild-type strain (Fig. 3B). Also, Δ*hrpG* still showed invasion ability into the fungus. This showed that RSSC does not use T3SS for this invasion. Altogether, the RSSC invasion mechanism into F. oxysporum was different from that of the M. rhizoxinica-R. microsporus interaction. The endoparasitic factors controlled by the *phc* QS system remain a mystery.

### EPS I is important for RSSC invasion into F. oxysporum chlamydospores.

**FIG 4:**
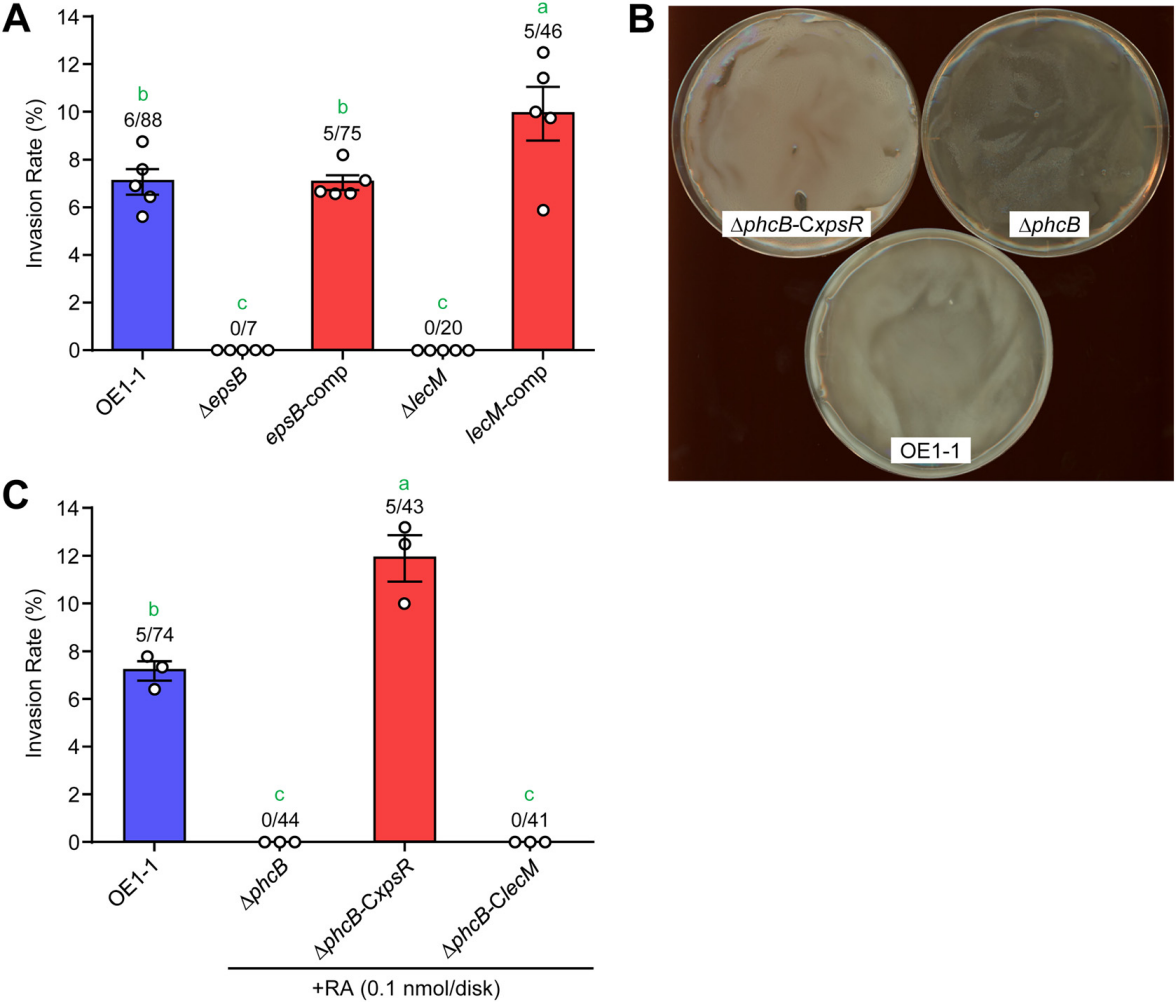
Effects of EPS I and LecM on the RSSC endoparasitism in F. oxysporum. (A) Invasion rates of Δ*epsB*, *epsB*-comp, Δ*lecM*, and *lecM*-comp into F. oxysporum chlamydospores. (B) The colonies of Δ*phcB*-C*xpsR*, Δ*phcB*, and OE1-1 on BG agar plates. EPS I quantities differed among strains (Δ*phcB* < OE1-1 < Δ*phcB*-C*xpsR*). (C) Invasion rates of Δ*phcB*, Δ*phcB*-C*xpsR*, and Δ*phcB*-C*lecM* into F. oxysporum chlamydospores. Ralstonin A (RA) was used to induce F. oxysporum chlamydospores. The error bars show the mean ± SEM (*n *= 3 or 5). The numbers above the bars show infected spores/total spores. The green letters above the bars show significant differences (*P < *0.05, Tukey’s test).

The production of EPS I (a major extracellular polysaccharide of RSSC) and LecM (an RS-IIL lectin) is also dependent on the activation of the *phc* QS system (31, 32, 37). As shown in Pseudomonas aeruginosa (41), EPS and lectin may comprise the extracellular matrix of RSSC cells. To investigate the importance of EPS I and LecM, we examined the invasion rates of Δ*epsB* (EPS I deficient) and Δ*lecM* (LecM deficient). These defective mutants were not parasitic on F. oxysporum, whereas the gene-complemented strains (*epsB*-comp and *lecM*-comp) were (Fig. 4A). We previously confirmed that the *phc* QS system in Δ*epsB* and Δ*lecM* does not function appropriately due to unveiled feedback regulation (42, 43). To investigate the true importance of EPS I and LecM, we complemented the production of these factors in Δ*phcB* (named them Δ*phcB*-C*xpsR* and Δ*phcB*-C*lecM*, respectively) and examined the invasion rates of these gene-complemented strains. Because XpsR is a transcriptional regulator for *eps* biosynthetic gene cluster in RSSC (44, 45), Δ*phcB*-C*xpsR* constitutively produced EPS I (Fig. 4B). The results of invasion assays showed that Δ*phcB*-C*xpsR* showed higher invasion rates than strain OE1-1 in the presence of ralstonin A, although Δ*phcB* and Δ*phcB*-C*lecM* could not invade the chlamydospores (Fig. 4C). Without ralstonin A addition, Δ*phcB*-C*xpsR* showed no invasion ability due to a lack of chlamydospore formation (Fig. S3). Thus, EPS I production is vital for RSSC entry into F. oxysporum chlamydospores.

### EPS I is important for the biofilm formation of RSSC cells on F. oxysporum hyphae.

**FIG 5:**
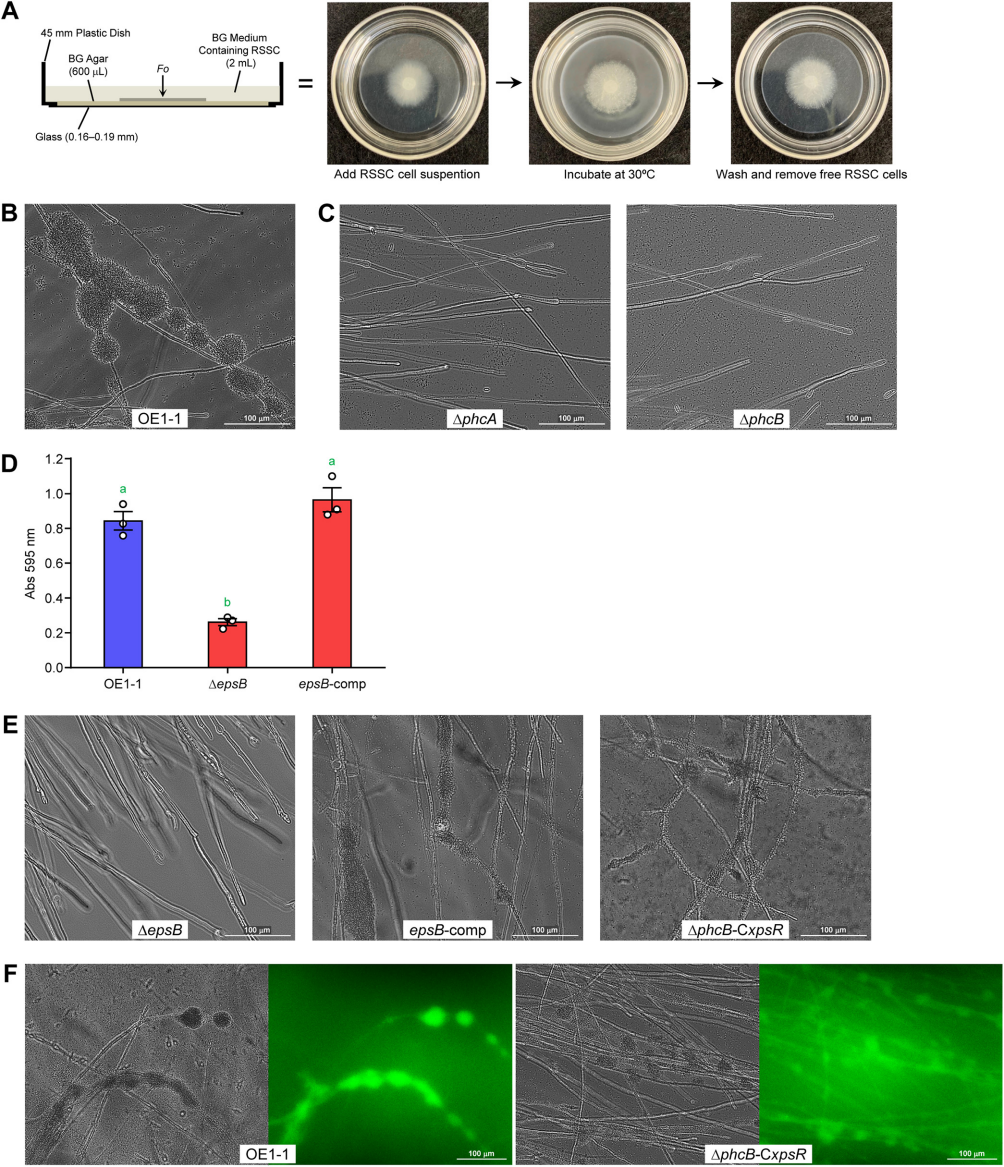
Importance of EPS I on RSSC biofilms’ formation on F. oxysporum hyphae. (A) A glass-bottomed dish assay to observe the interaction between RSSC and F. oxysporum. Images of the assay plate (left) and the assay method (right) are shown. (B) Biofilms of OE1-1 on F. oxysporum hyphae. RSSC cells form biofilms like a grape cluster on the hyphae. (C) Photos of coculturing of F. oxysporum with Δ*phcA* and Δ*phcB*. These mutants did not form biofilms. (D) Biofilms of OE1-1, Δ*epsB*, and *epsB*-comp on a plastic plate. The vertical axis represents the amount of biofilm as the absorbance of the recovered crystal violet. The error bars show the mean ± SEM (*n *= 3). The green letters above the bars show significant differences (*P < *0.05, Tukey’s test). (E) Photos of coculturing of F. oxysporum with Δ*epsB*, *epsB*-comp, and Δ*phcB*-C*xpsR*. The biofilm formation was observed in *epsB*-comp and Δ*phcB*-C*xpsR*. (F) DBA-FITC staining of the biofilms of OE1-1 and Δ*phcB*-C*xpsR*. For each pair of images, the bright field image is on the left, and the fluorescence image is on the right.

To investigate how EPS I is involved in the RSSC parasitism, we observed the interaction between OE1-1 and F. oxysporum during a coculture assay in a glass-bottomed dish (Fig. 5A) and looked for event(s) that EPS I might be involved in. We found that RSSC cells formed biofilm-like structures on the fungal hyphae before inducing the chlamydospores (Fig. 5B). This implied that the bacterial biofilms on the host may be involved in RSSC-F. oxysporum interaction. The QS-deficient mutants Δ*phcA* and Δ*phcB* did not adhere to the mycelia of F. oxysporum, nor did they form biofilms there (Fig. 5C). Thus, the cell attachment and biofilm formation, regulated by the *phc* QS system (22, 38), may be essential traits for the RSSC parasitism on the host hyphae.

The attachment of RSSC cells to solid surfaces (such as plastic) and the formation of biofilms there are dependent on the production of EPS I (Fig. 5D) (46). However, there was no knowledge of whether EPS I is involved in the RSSC adhesion to fungal mycelia and biofilm formation. The influence of *epsB* deletion or complementation was evaluated by coculture assays with F. oxysporum. Although Δ*epsB* did not form the biofilm on F. oxysporum hyphae, it did form the biofilm after the complementation of the *epsB* gene (Fig. 5E). Notably, Δ*phcB*-C*xpsR* showed the ability to form biofilms at a high frequency on F. oxysporum hyphae, although the biofilm was thinner than that of the wild-type strain. Therefore, we expected that even with increased EPS I levels, the biofilms would not mature due to the absence of other factors (such as LecM) in Δ*phcB*-C*xpsR*. Additionally, when the fluorescent staining of EPS I was conducted using Dolichos biflorus agglutinin (DBA; a lectin with specificity for α-linked *N*-acetylgalactosamine)-fluorescein isothiocyanate (FITC) (47), FITC-derived fluorescence was observed in OE1-1 and Δ*phcB*-C*xpsR* biofilms (Fig. 5F). Due to the increased staining and washing operations, the biofilms were somewhat more broken than those in Fig. 5B and E. This showed that EPS I is a major matrix component of RSSC biofilms on F. oxysporum hyphae. EPS I production was definitely found to be essential for the biofilm formation of RSSC cells on F. oxysporum hyphae.

### EPS I quality and quantity are important for RSSC endoparasitism.

**FIG 6:**
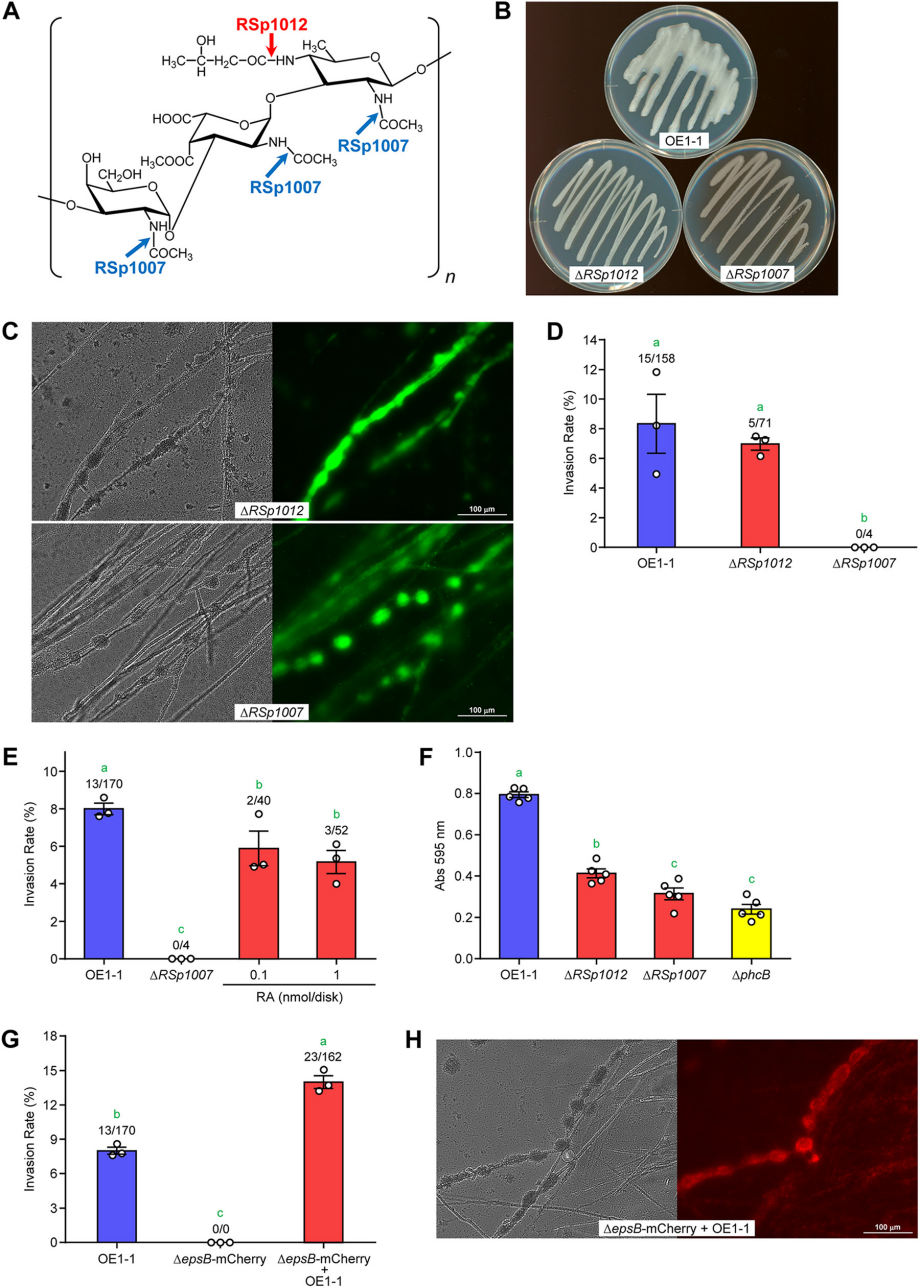
Importance of EPS I structure to RSSC endoparasitism in F. oxysporum and Δ*epsB* endoparasitism recovery by EPS I from the OE1-1 strain. (A) EPS I structure produced by RSSC strains. RSp1012 and RSp1007 are involved in transfer reactions at the positions shown by the arrows. (B) Photo of OE1-1, Δ*RSp1012*, and Δ*RSp1007* grown on BG agar plates. Colony liquidity derived from EPS I is significantly reduced in Δ*RSp1012* and Δ*RSp1007*. (C) Biofilms of Δ*RSp1012* and Δ*RSp1007* on F. oxysporum hyphae. SYTO9 was used to stain bacterial cells. For each pair of images, the bright field image is on the left, and the fluorescence image is on the right. (D) Invasion rates of OE1-1, Δ*RSp1012*, and Δ*RSp1007* into F. oxysporum chlamydospores. (E) Effect of ralstonin A (RA) on Δ*RSp1007* invasion into F. oxysporum chlamydospores. (F) OE1-1, Δ*RSp1012*, Δ*RSp1007*, and Δ*phcB* biofilms on plastic plates. The vertical axis represents the amount of biofilm as the absorbance of the recovered crystal violet. (G) Invasion rates of OE1-1, Δ*epsB*-mCherry, and Δ*epsB*-mCherry cocultured with OE1-1 into F. oxysporum chlamydospores. (H) Biofilm formation by OE1-1 and Δ*epsB*-mCherry on F. oxysporum hyphae. For each pair of images, the bright field image is on the left, and the fluorescence image is on the right. The error bars show the mean ± SEM (*n *= 3 or 5). The numbers above the bars show infected spores/total spores. The green letters above the bars show significant differences (*P < *0.05, Tukey’s test).

We predicted that the EPS I-dependent biofilm formation of RSSC cells may be necessary for their parasitism on F. oxysporum. To examine this, we tested the parasitic potential of mutant strains lacking the enzymes involved in EPS I biosynthesis, i.e., to change the substructure of EPS I (Fig. 6A). RSp1012, a LpxA-like protein, transfers a 3-hydroxybutanoate to the 2,4-diamino-2,4,6-trideoxygalactose moiety in EPS I (48). Δ*RSp1012* produced the EPS I that differed in appearance from that of the wild-type strain (Fig. 6B). The mutant formed a more brittle biofilm than OE1-1 on F. oxysporum hyphae, and their thickness became thinner (Fig. 6C, upper). Δ*RSp1012* parasitized the fungus at almost the same rate as the wild-type strain (Fig. 6D). The deletion of the gene *RSp1007* encoding the enzyme that transfers the acetate to amino sugars (Fig. 6A) (49) resulted in reduced EPS production and no invasion of the mutant into F. oxysporum chlamydospores (Fig. 6B and D). Ralstonin production was reduced in Δ*RSp1007* (Fig. S4). This may be obtained from the feedback inhibition observed in Δ*epsB* and Δ*lecM* (42, 43). Thus, the invasion assay of Δ*RSp1007* with ralstonin A was conducted. Certain parasitism recovery rates were observed, but not to wild-type strain levels (Fig. 6E). In a glass-bottomed dish assay, Δ*RSp1007* formed a fragile biofilm on F. oxysporum mycelium, and the frequency of its formation decreased slightly (Fig. 6C, lower). In a biofilm test using a plastic assay plate, both mutants formed slightly more biofilms than Δ*phcB*, and the amounts were greatly reduced compared to OE1-1 (Fig. 6F). The biofilm on plastic was probably more influenced by the quality and quantity of EPS I. Combined, we showed that EPS I, which acts somewhat as a cell-to-cell adhesive, is essential for RSSC to parasitize F. oxysporum and appropriately drive QS circuits.

### Δ*epsB* cells use EPS I produced by wild-type RSSC cells.

To examine whether EPS I-deficient cells used EPS I generated by RSSC cells, we mixed Δ*epsB* and OE1-1 and examined Δ*epsB* parasitism on F. oxysporum. To distinguish between Δ*epsB* and wild-type cells, mCherry protein was expressed in Δ*epsB* (named Δ*epsB*-mCherry). mCherry-derived fluorescence was observed in the F. oxysporum chlamydospores from coculturing experiments, confirming that Δ*epsB*-mCherry is taken up by the spores (Fig. 6G). Furthermore, the invasion rate was higher than that of the wild-type strain. Next, we examined whether Δ*epsB* was incorporated into the biofilms of OE1-1 during a coculture assay. Δ*epsB*-mCherry, along with the wild-type strain, formed mature biofilms on F. oxysporum hyphae, although the uptake ratio of the mutant varied from biofilm to biofilm (Fig. 6H). When the mixture of Escherichia coli DH5α-mCherry and OE1-1 was cocultured with F. oxysporum, the E. coli strain was not incorporated into the RSSC biofilms or the F. oxysporum chlamydospores (Fig. S5). These results indicated that Δ*epsB* could invade F. oxysporum chlamydospores, possibly by using surrounding EPS I (from OE1-1) as a biofilm matrix, but this property could not be reproduced in other bacteria; e.g., E. coli.

### Ralstonin is also involved in the biofilm formation of RSSC on F. oxysporum hyphae.

**FIG 7:**
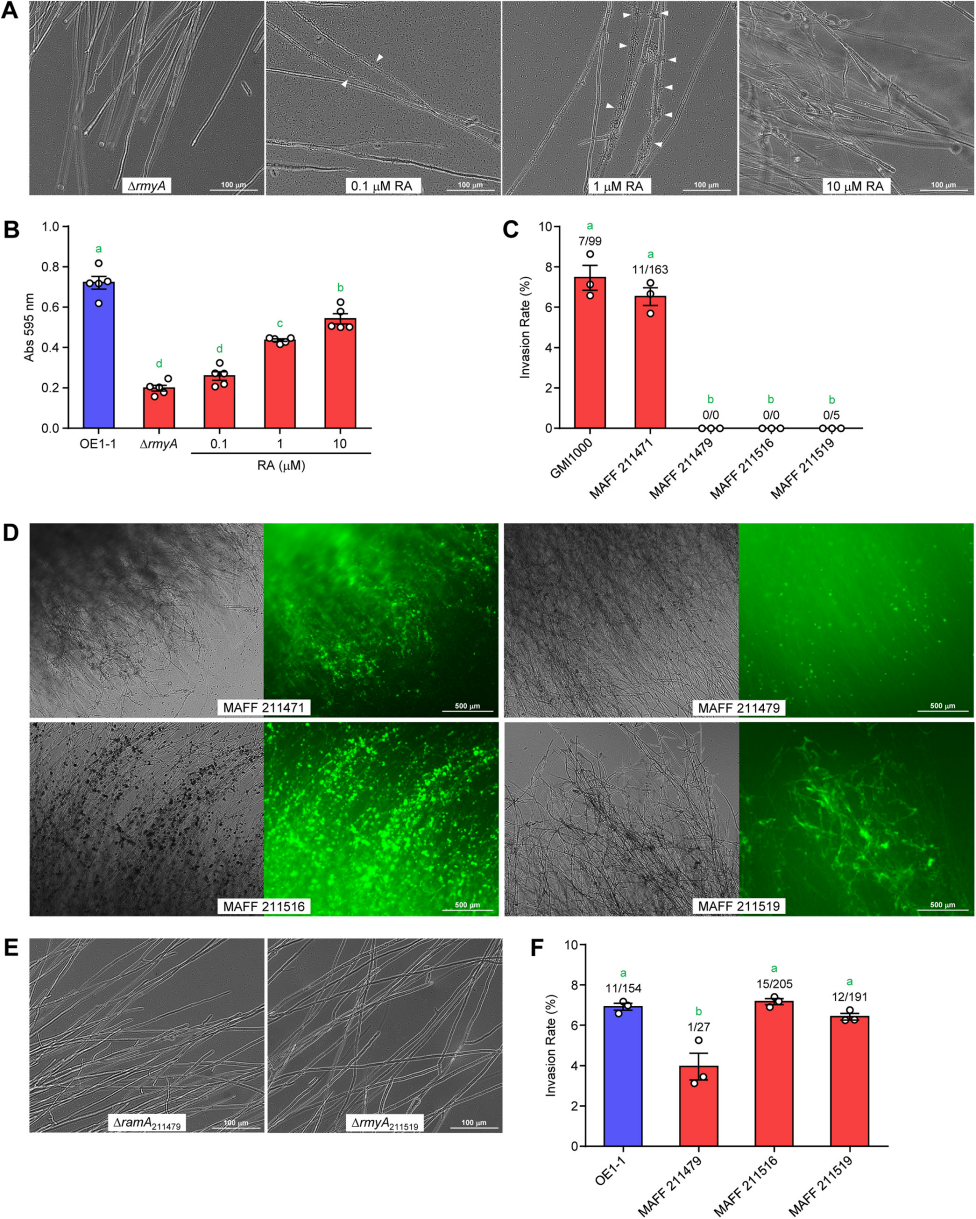
Importance of lipopeptides on RSSC endoparasitism in F. oxysporum. (A) Ralstonin-dependent recovery of biofilm formation in Δ*rmyA*. White arrowheads indicate biofilms. Ralstonin A (RA) supplement at 10 μM caused the chlamydospore formation of F. oxysporum. (B) Biofilm formation of Δ*rmyA* on a plastic plate and its response to ralstonin A. The vertical axis represents the amount of biofilm as the absorbance of the recovered crystal violet. (C) Invasion rates of the wild-type RSSC strains into F. oxysporum chlamydospores. GMI1000 and MAFF 211471 produce ralstonins, MAFF 211479 and MAFF 211516 produce ralstoamides, and MAFF 211519 produces ralstopeptins. (D) Biofilms of MAFF strains on F. oxysporum hyphae. SYTO9 was used to stain the bacterial cells. For each pair of images, the bright field image is on the left, and the fluorescent image is on the right. (E) Lack of biofilm formation ability in Δ*ramA*_211479_ and Δ*rmyA*_211519_ on F. oxysporum hyphae. (F) Acquisition of endoparasitic ability of MAFF strains by coculturing with OE1-1. Only OE1-1 is a positive control. The error bars show the mean ± SEM (*n *= 3). The numbers above the bars show infected spores/total spores. The green letters above the bars show significant differences (*P < *0.05, Tukey’s test).

According to reports, lipopeptides are involved in the biofilm formation of Gram-negative bacteria as biosurfactants (50, 51). Thus, we investigated whether ralstonins are responsible for the biofilm formation in RSSC cells. Δ*rmyA* did not form biofilms on the hyphae of F. oxysporum, and biofilm formation was partially restored upon ralstonin A addition (0.1 and 1 μM) (Fig. 7A). However, no biofilm was observed at 10 μM; instead, chlamydospores formed faster due to increased ralstonin A concentrations. Regarding the biofilm formation assay using a plastic assay plate, Δ*rmyA* resulted in less biofilm than the wild-type strain, and ralstonin addition rescued the biofilm formation of Δ*rmyA* (Fig. 7B). In a plate assay, 10 μM ralstonin A did not inhibit biofilm formation. These results suggested that high ralstonin A concentrations and/or formed chlamydospores may be inhibitory toward biofilm formation on F. oxysporum. Altogether, ralstonins have essential roles in RSSC biofilm formation on F. oxysporum mycelia.

Some RSSC strains produce ralstopeptins or ralstoamides, which have different structures from ralstonins (Fig. S6) (52, 53). RSSC lipopeptides other than ralstonins did not show strong chlamydospore-inducing activity (53). We speculated whether they were involved in RSSC biofilm formation on F. oxysporum mycelia. To investigate this, other RSSC strains producing ralstonins, ralstoamides, or ralstopeptins were cocultured with F. oxysporum. MAFF 211471 and GMI1000, which produce ralstonins, exhibited an invasion rate close to that of OE1-1, but ralstoamide-producing strains MAFF 211479 and MAFF 211516 and the ralstopeptin-producing strain MAFF 211519 failed to establish the endoparasitism (Fig. 7C). This may be because little or no chlamydospore formation occurred in the assays with MAFF 211479, MAFF 211516, and MAFF 211519. These MAFF strains formed biofilms on the mycelia in a glass-bottomed dish assay, although their ability to do so differed (Fig. 7D). The biofilms were easily collapsed by the washing process compared with strain OE1-1. Δ*rmyA*_211519_ (a ralstopeptin-deficient mutant of MAFF 211519) (53) and Δ*ramA*_211479_ (a ralstoamide-deficient mutant of MAFF 211479) (52) were found to have lost the ability to form biofilms (Fig. 7E). Thus, ralstoamides and ralstopeptins also contribute to biofilm formation. When these MAFF strains were tested together with OE1-1 for parasitism into F. oxysporum, MAFF 211516 and MAFF 211519 exhibited invasion rates comparable to the OE1-1 strain (Fig. 7F). It was likely that ralstonin A from the OE1-1 strain induced F. oxysporum chlamydospores, which ultimately allowed MAFF strains to invade spores. MAFF 211479 appeared to exhibit low invasion ability, even if OE1-1 was present. Just as RSSC virulence on plants varies from strain to strain, so too can its ability to parasitize F. oxysporum vary from strain to strain. EPS I quality and quantity in MAFF strains may differ from the OE1-1 strain. Overall, lipopeptides produced by RSSC strains were important for biofilm formation on F. oxysporum mycelia—again suggesting that ralstonins were essential for this endoparasitism.

### RSSC infection kills F. oxysporum chlamydospores.

**FIG 8:**
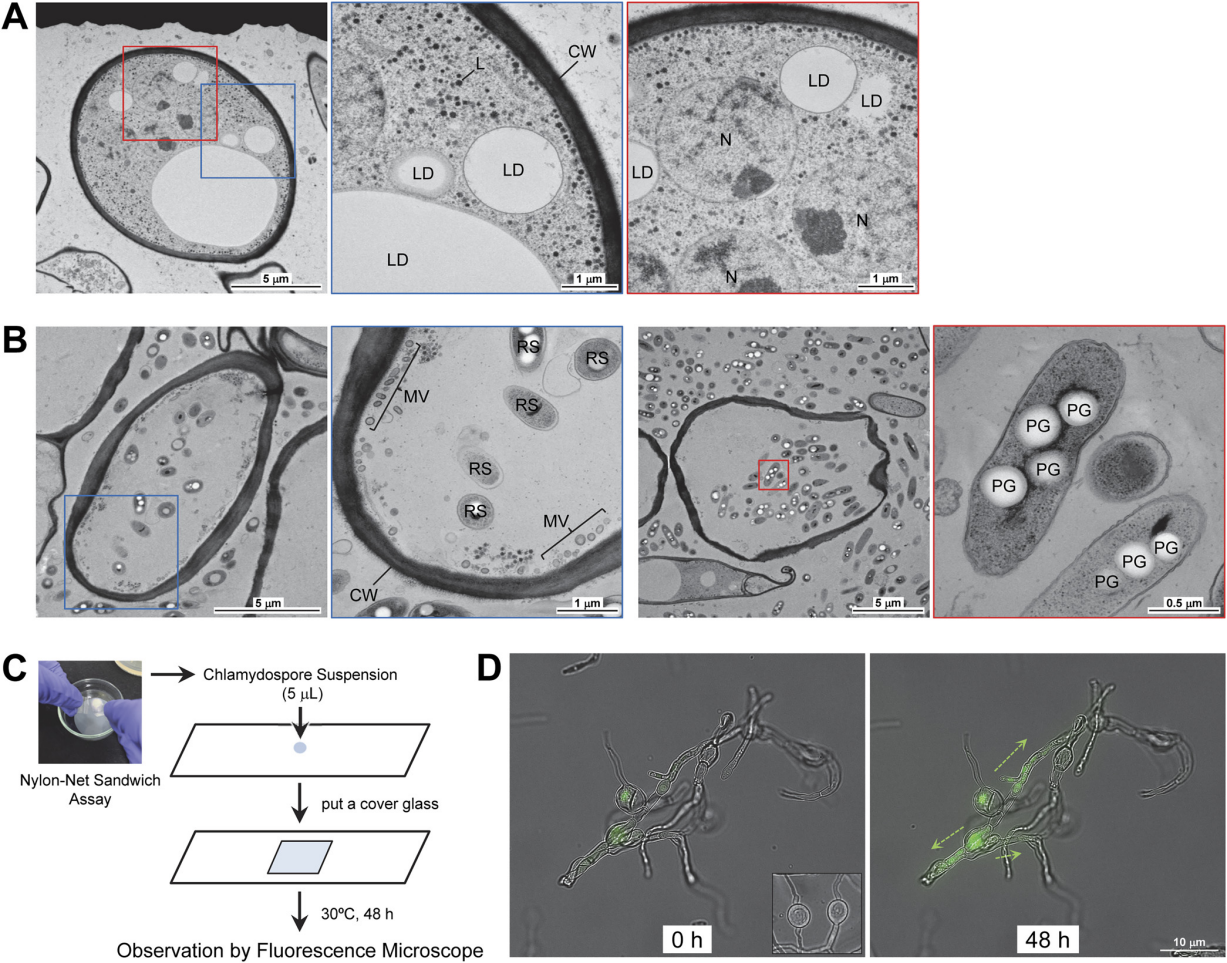
Microscopic observation of intact and infected F. oxysporum chlamydospores. (A) Transmission electron microscopy (TEM) images of F. oxysporum chlamydospores induced by ralstonin A treatment. Enlarged views of the areas framed in blue and red are shown on the right. (B) TEM images of F. oxysporum chlamydospores infected with OE1-1. Enlarged views of the areas framed in blue or red are shown on the right. (C) Preparation of a sample for the observation of RSSC behavior in F. oxysporum chlamydospores. (D) Fluorescence observation of infected chlamydospores immediately after collection and 48 h later. The inset shows the chlamydospores induced by ralstonin A. CW, cell wall; L, liposome; LD, lipid droplet; MV, membrane vesicle; N, nuclear; PG, polyhydroxyalkanoate granule; RS, RSSC cell.

It was expected that TEM observation of chlamydospores infected by RSSC may provide some insight into the invasion mechanism and the postinvasion behaviors of RSSC. The TEM observation of chlamydospores induced by ralstonin A showed that the structures were almost identical to those previously reported (Fig. 8A) (54, 55). Particularly, the cell wall was thick, and large lipid droplets, nuclei, and ribosomes were observed in the spores. The spores were histologically confirmed to be F. oxysporum chlamydospores. Next, F. oxysporum chlamydospores infected with RSSC were observed by TEM. TEM observation of the chlamydospores formed after 3 days of incubation of F. oxysporum with OE1-1 showed that the spores had disrupted cell membranes, which changed to membrane vesicles (Fig. 8B). Furthermore, organelles such as lipid droplets and nuclei disappeared, and some to many RSSC cells were observed in the chlamydospores. Bigger polyhydroxyalkanoate granules (56, 57) were observed in the RSSC cells, suggesting that the chlamydospore contents may be used as nutrients. These observations showed that these chlamydospores were killed by the RSSC’s infection and growth.

We examined whether the cell death of F. oxysporum chlamydospores, as observed in the TEM analysis, is also observed in the unfixed chlamydospores after RSSC infection. We used a method that allowed us to observe the parasitized spores under high magnification by a fluorescence microscope (Fig. 8C). The F. oxysporum spores infected with OE1-1 were collected and allowed to grow on a glass slide. The OE1-1-derived GFP fluorescence was observed over time. The RSSC cells were found to grow within infected chlamydospores and migrate into nearby hyphae (Fig. 8D). Notably, what appeared to be cell membranes within the chlamydospores deviated from the cell walls. Alternatively, the chlamydospores induced by ralstonin A were almost spherical and showed no signs of separation of the cell membranes from the cell walls (Fig. 8D, inset). Therefore, we concluded that the chlamydospores infected by RSSC cells were dead, and the bacteria could migrate to the surrounding mycelia.

## DISCUSSION

This study demonstrates that the *phc* QS system is important for RSSC to invade F. oxysporum. Δ*phcB* lost the abilities to produce ralstonins and to invade F. oxysporum chlamydospores, and these defects were restored by the exogenous 3-OH MAME but not by ralstonin A. Analysis of the gene-deletion and -complemented mutants showed that, in addition to ralstonins, EPS I is an important parasitic factor. Moreover, EPS I and ralstonins are required for RSSC biofilm formation on F. oxysporum hyphae. Biofilm formation is essential for RSSC endoparasitism in F. oxysporum.

**FIG 9:**
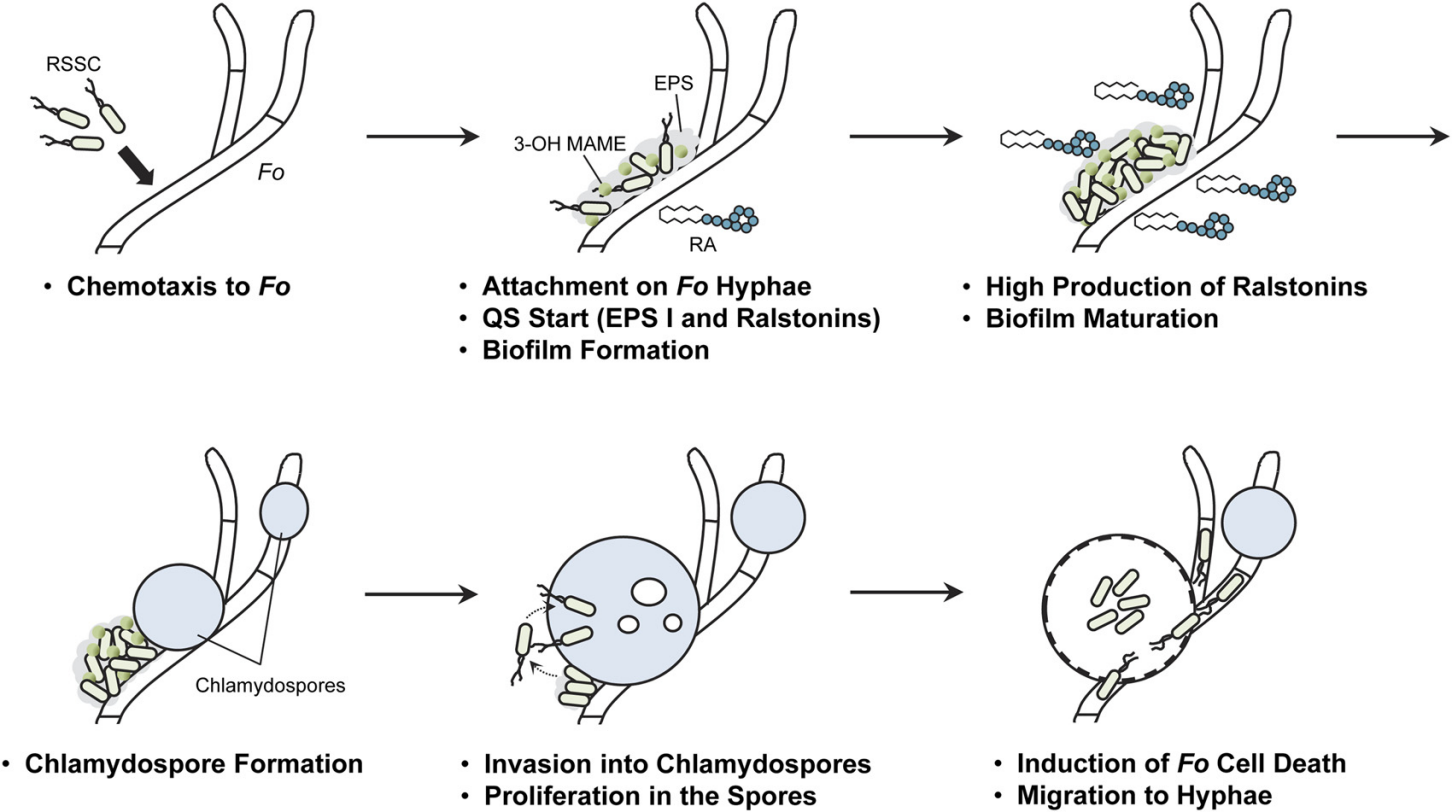
Proposed mechanism of RSSC endoparasitism in F. oxysporum. *Fo*, F. oxysporum; RA, ralstonin A.

Based on these results, we proposed a mechanism by which RSSC cells parasitize into F. oxysporum via the chlamydospores induced (Fig. 9). RSSC cells exhibit positive chemotaxis toward F. oxysporum hyphae and migrate toward them. This can be seen in the postcoculture photo in Fig. 5A. After adherence, RSSC cells begin to drive the *phc* QS system. Consequently, they produced EPS I, ralstonins, and simultaneously formed biofilms. When bacterial cells increased and the QS system is fully activated, the biofilm matures and the production of ralstonins becomes high. Once F. oxysporum chlamydospores are formed, RSSC cells begin to detach from the biofilms, and some invaded spores. After invasion, RSSC cells proliferated, presumably using spore contents as nutrients. The infection killed chlamydospores, and RSSC cells started migrating to surrounding hyphae.

The *phc* QS of RSSC strains is a critical system for parasitism in plants that is properly activated at each step of infection, allowing them to invade and multiply within the hosts (18, 19). For example, the *phc* QS system regulates not only the production of the virulence factors (e.g., EPS I and plant-cell-wall-degrading enzymes) necessary for plant infection but also causes the metabolic changes to adapt the infection stages (37, 38). We found that in the RSSC endoparasitism on F. oxysporum, the *phc* QS system regulates biofilm formation on the hyphae and induces the chlamydospore formation of F. oxysporum. The production of ralstonins and EPS I, key in these events, is under the QS control. Alternatively, T2SS and T3SS, also regulated by the *phc* QS system (37, 38), were not vital in this endoparasitism. This was different from the invasion strategy of M. rhizoxinica into the hyphae of R. microsporus (34). HrpG was reported to be partially involved in producing the QS-dependent factors (such as EPS I) (40), which may explain the slightly reduced tendency for endoparasitism observed in the Δ*hrpG* strain. Therefore, corporative cell-cell activities regulated by the *phc* QS system play vital roles in this lethal interaction with the fungus.

Biofilm formation by bacteria on fungal mycelia has been sporadically reported (58–60). However, we were the first to discover that biofilms are essential for bacterial endoparasitism within fungi. Particularly, EPS I and ralstonins were found to be essential for RSSC to form biofilms on F. oxysporum hyphae. The overexpression of *xpsR* increased EPS I production and biofilm formation frequency. This resulted in a significant increase in the invasion rate of the recombinant compared with that of the wild-type strain. The EPS I around the bacterial cells was expected to increase the affinity of RSSC cells to F. oxysporum hyphae and wrap the aggregated cells there. The structure and amount of EPS I appear to be essential for the frequency and maturity of biofilms. Galactosaminogalactan was reported to contribute to the self-aggregation of Aspergillus oryzae hyphae (61, 62). A similar mechanism might be involved in increasing the affinity of EPS I for F. oxysporum hyphae. RSSC lipopeptides appeared to function as a biosurfactant and contribute to the biofilm formation. We cannot say for certain about the structural specificity of RSSC lipopeptides required for biofilm formation. Even mutants deficient in EPS I and/or lipopeptide production showed endoparasitism into F. oxysporum in the presence of the wild-type OE1-1. These factors are unlikely to be involved in invasion processes after biofilm formation and chlamydospore induction.

Our analyses showed that RSSC lipopeptides are essential factors in deciding which RSSC strains can be endoparasites for F. oxysporum. The ralstonin-producing strains (OE1-1 and GMI1000) will likely be the best endoparasites among the tested strains. The ralstoamide-producing strains MAFF 211479 and MAFF 211516 and the ralstopeptin-producing strain MAFF 211519 were not parasitic on F. oxysporum. When ralstonins were supplied from the OE1-1 strain, the MAFF strains also parasitized. Recently, we reported a size reduction in PKS-NRPS genes (*rmyA*/*rmyB* and *ramA*) in several RSSC strains (53). When combined with data from this study, RSSC strain endoparasitism into fungi may be easily lost due to mutations in PKS-NRPS genes. Compared with pathogenicity in plants, endoparasitism in fungi may be an unstable trait for RSSC strains. A bioinformatic tool search did not find any bacteria (other than Ralstonia) producing a lipopeptide with a chemical structure similar to ralstonins. Fengycin A has chlamydospore-inducing activity against F. oxysporum and restores the parasitic potential of Δ*rmyA*, but the producer Bacillus sp. was not reported to invade F. oxysporum cells (54, 63). In a previous study, a lipopeptide was reported to function as a biosurfactant to facilitate endoparasitism in a host fungus (64). However, in this case, chlamydospore formation was not involved in endoparasitism. The discovery of further bacteria that can induce chlamydospores and endoparasitism against fungi may help to investigate whether the mechanism of RSSC’s endoparasitism is more ubiquitous.

Although our knowledge of the interaction between RSSC and F. oxysporum has deepened, how RSSC invades the F. oxysporum chlamydospores was not entirely clear in this study. According to our analysis, T2SS and T3SS are not involved in this endoparasitism. This was not our initial expectation. Some lipopeptides act as cell toxins by aggregating to form pores in plant cell membranes to facilitate nutrient exit or to disturb the ion balance inside in the cell (65–67). Although ralstonin A treatment induced chlamydospore formation in F. oxysporum, such membrane disruption could not be seen from TEM and microscopic analyses. Instead, many mycelia changed to chlamydospores, and the cell membranes (or walls) became thicker. Thus, it is unknown during what period the cell membrane is disrupted. Endocytosis is an example of a mechanism of bacterial uptake by fungal cells (68). However, although many sections were observed by TEM, so far, no images indicated the involvement of endocytosis. The possibility remains that we missed getting an image of the moment. To elucidate this mechanism, it will be important to examine microscopic observations at finer time intervals, as well as the responses of F. oxysporum to the RSSC’s parasitism.

While this study was being prepared, a new paper on the parasitism of RSSC into a fungus was reported by Keller’s group (63). There, the importance of chlamydospore induction by ralstonin A was shown using a different approach compared to this study. Additionally, several Gram-negative bacteria acted as “hitchhikers” with RSSC cells and could invade the chlamydospores of the different host Aspergillus flavus. They reported that this event could be triggered simply by treating ralstonin A instead of RSSC cells. This is inconsistent with this study’s experimental results, in which E. coli failed to infect F. oxysporum by simultaneous inoculation with wild-type RSSC cells. Even RSSC cells cannot invade F. oxysporum chlamydospores if QS or related genes are deficient. It did not recover with ralstonin alone. Likely, the difference in the hosts (F. oxysporum versus A. flavus) has a significant impact. Additionally, as confirmed in the M. rhizoxinica-R. microsporus system, RSSC cell surface components, such as LPS, may be involved in recognition by F. oxysporum (14). However, this mechanism is currently unclear and warrants further study.

What advantages would RSSC obtain by parasitizing the fungus? RSSC strains must continue to survive in the soil even without a suitable plant host. There could be a situation in which RSSC cells are present in the soil of a tomato field, but there are no tomato plants nearby to be infected. Fungi may be more ubiquitous, so the endoparasitism into fungi may be a good survival strategy for RSSC strains. It might be able to reach the host plants through fungal mycelia. As Keller’s group reported (63), endoparasitism in fungal hosts may confer resistance to environmental stresses on RSSC cells. Of course, the acquisition of stress tolerance is probably an important aspect of this endoparasitism. However, given the possibility that RSSC cells assimilate nutrients to the point of killing F. oxysporum cells, such parasitism within fungi may be a survival strategy in the soil. The relationship may vary depending on the type of host. Since both F. oxysporum and RSSC infect tomato plants (69, 70), we are interested to see how this endoparasitism will affect the emergence of these diseases.

In terms of pest control, we need better to understand the behaviors of RSSC cells in the soil and suggest appropriate control methods. There might be multiple interactions that we do not yet know about, such as RSSC parasitism and symbiosis with other organisms, some of which kill the host and some of which keep the host alive. Through previous and current studies (22), we have reported that the *phc* QS system controls the phytopathogenic and endofungal parasitic behaviors of RSSC. Thus, the *phc* QS system should be an attractive control target. If the QS system could be controlled by chemicals or microbes, it may be possible to prevent bacterial wilt disease before it develops and facilitate host plant recovery. We plan to examine the effects of QS inhibitors (such as *phc* quorum sensing inhibitors [PQIs]) (30) on this endoparasitism in detail.

Conclusively, we found a QS-dependent invasion of RSSC into F. oxysporum. We not only have confirmed the importance of ralstonins as F. oxysporum chlamydospore inducers but have also confirmed that EPS I production and biofilm formation are cleverly regulated by the *phc* QS system during the development of endoparasitism on F. oxysporum chlamydospores. The RSSC infection is likely a means of killing the F. oxysporum spores and obtaining nutrition. Consequently, RSSC appears to allow free movement within F. oxysporum. RSSC strains may be the phytopathogenic bacteria that have evolved to fit field conditions more cleverly than we imagine.

## MATERIALS AND METHODS

### Nylon net sandwich assay.

GFP-expressing RSSC strains were inoculated into 2× BG medium (2 mL) containing kanamycin (50 μg/mL) and cultured overnight by shaking. A nylon net filter (11 μm, 47 mm, Merck) was placed on the surface of a BG agar medium (25 mL in 90-mm plastic dish) containing kanamycin (100 μg/mL). After the B medium (50 μL) was added to the top of the net, another nylon net (20 μm, 47 mm, Merck) was placed on top. RSSC cell suspension (5 μL) and F. oxysporum NBRC 31213 spore suspension (5 μL) were spotted on the top net, 15 mm apart from each other. The plate was incubated at 30°C for 3 days. The top net filter was removed from the medium and washed thoroughly with MilliQ water to remove the bacteria cells. Chlamydospores were collected by rubbing the surface using a cover glass. The collected chlamydospores were washed with MilliQ water (0.5 mL) and observed under a fluorescence microscope (BZ-X810, Keyence). The invasion rate (% = chlamydospores with RSSC/total chlamydospores × 100) per condition was calculated from three to five dishes.

### Biofilm formation assay.

RSSC strains were incubated in CPG medium (2 mL) at 30°C overnight. The cultures were transferred to 2-mL tubes and centrifuged at 15,000 × *g* for 3 min. The old media were discarded, and the bacterial cells were suspended in CPG medium (1 mL), and the optical density at 600 nm (OD_600_) was adjusted to 0.1. New CPG medium (95 μL) and bacterial cell suspension (5 μL) were dispensed into each well of a 96-well microtiter plate (Thermo Fisher Scientific). The plates were sealed with Breathe-Easy membrane (Sigma-Aldrich) and statically incubated at 30°C for 20 h. To the culture medium, 1% (wt/vol) crystal violet solution (25 μL) was added and allowed to stand at room temperature for 25 min, and then excess crystal violet was removed with a pipette. The well was then washed twice with MilliQ water (200 μL). Ethanol (200 μL) was added and allowed to stand for 10 min to extract the crystal violet. The solution was transferred to a flat-bottomed 96-well plate (Iwaki), and the absorbance at a wavelength of 595 nm was measured using a plate reader (Multiskan FC, Thermo Fisher Scientific).

### Glass-bottomed dish assay.

Hot BG agar medium (600 μL, 1% [wt/vol] agar) is dispensed into a glass-bottomed dish (Matsunami). After cooling, F. oxysporum spore suspension (2 μL) was spotted on the surface of BG agar. The dish was incubated for 36 h at 30°C. The RSSC cell suspension in BG medium (OD_600_ = 0.25, 2 mL) was pipetted onto the surface of the F. oxysporum colony in the dish. The BG medium was removed by pipetting after incubation for 9 or 24 h at 30°C. By washing with MilliQ water (1 mL) twice, the excess RSSC cells were discarded. If necessary, a staining operation was added before washing. DBA-FITC (Vector Laboratories) and SYTO9 (Thermo Fisher Scientific) were used to stain EPS I and bacterial cells, respectively. The dish was observed using a fluorescence-inverted microscope (BZ-X810).

### Construction of gene-deletion mutants.

A synthetic gene was prepared by fusing 450 bp upstream and 450 bp downstream of the target gene (*RSp0275*, *RSp0924*, *RSc0818*, *RSp0138*, *RSp0161*, *gspD*, *RSp1012*, and *RSp1007*) (Eurofins Genomics). The fused gene was released from the provided vector by restriction enzyme digestion and introduced into the pK18mobsacB vector (Table S1). This plasmid was electroporated into RSSC cells (OD_600_ = 0.6, in 10% [wt/vol] glycerol), and the cells were incubated in SOC medium (1 mL, Sigma-Aldrich) for 6 h at 30°C. After this, kanamycin-resistant (50 μg/mL) and sucrose-sensitive (10% [wt/vol]) recombinants were selected. After 4 h of incubation in SOC medium, kanamycin-sensitive (50 μg/mL) and sucrose-resistant (10% [wt/vol]) recombinants were reselected. Finally, targeted deletions were confirmed by PCR. Both Δ*egl* and Δc*bhA* mutants were generous gifts from Hikichi (Kochi University) (71).

### Construction of gene-complemented mutants.

Plasmids for gene complementation studies (Table S1) were prepared by PCR-based cloning or from synthetic genes (Eurofins Genomics). Plasmids were electroporated into RSSC cells, and kanamycin-resistant (50 μg/mL) recombinants were selected. Target gene introductions were confirmed by PCR.

### Isolation of ralstonins A and B.

OE1-1 cells grown in B medium for 6 h was spread on 1,000 BG agar plates, and the plates were incubated at 30°C for 3 days. BG agar was cut into small pieces and soaked in acetone (20 L) for 2 h. The acetone extract was collected by filtration, and the agar residue was soaked again in acetone (20 L) for 2 h. The combined extracts were evaporated to remove acetone, then extracted twice in ethyl acetate (EtOAc), and dried over Na_2_SO_4_. The EtOAc extracts were concentrated and chromatographed on a silica gel column, and eluted stepwise with solvents of increasing polarity from *n*-hexane to EtOAc and then from EtOAc to methanol (MeOH). The fractions containing ralstonins (60% and 100% MeOH eluates) were subjected to high-pressure liquid chromatography (HPLC) with an InertSustain C18 column (250 mm × 10 mm, 5 μm) at a flow rate of 4 mL/min with 45% acetonitrile in 0.1% aq. trifluoroacetic acid to yield ralstonins A (13 mg) and B (1.0 mg).

### TEM observation.

F. oxysporum chlamydospores induced by ralstonin A or by coculturing with strain OE1-1 were used. Ultrastructural analyses of the chlamydospores were conducted by Tokai-EMA (Japan).

### Statistical analysis.

The mean values and standard error of the mean (SEM) were calculated using GraphPad Prism 6.0. The levels of significance were evaluated using one-way analysis of variance (ANOVA), followed by Tukey’s or Dunnett’s multiple-comparison tests by GraphPad Prism 6.0.
