# Supplementary material for: Quorum Sensing-Dependent Invasion of Ralstonia solanacearum into Fusarium oxysporum Chlamydospores
Source: Microbiol Spectr. 2023 Jun 27;11(4):e00036-23. doi: 10.1128/spectrum.00036-23 (PMC10433826; doi:10.1128/spectrum.00036-23)
Supplement: Supplemental file 1 — Supplemental material. Download spectrum.00036-23-s0001.pdf, PDF file, 1.0 MB [file spectrum.00036-23-s0001.pdf]

Supporting Material

Quorum Sensing-Dependent Invasion of *Ralstonia solanacearum* into *Fusarium oxysporum* Chlamydospores

Chiaki Tsumori,[†,a] Shoma Matsuo,[a] Yuta Murai,[a] and Kenji Kai[†,*,a]

[†] These authors contributed equally to this work.

[a] Graduate School of Agriculture, Osaka Metropolitan University, 1-1 Gakuen-cho, Naka-ku, Sakai, Osaka 599-8531, Japan

E-mail: kenjikai@omu.ac.jp

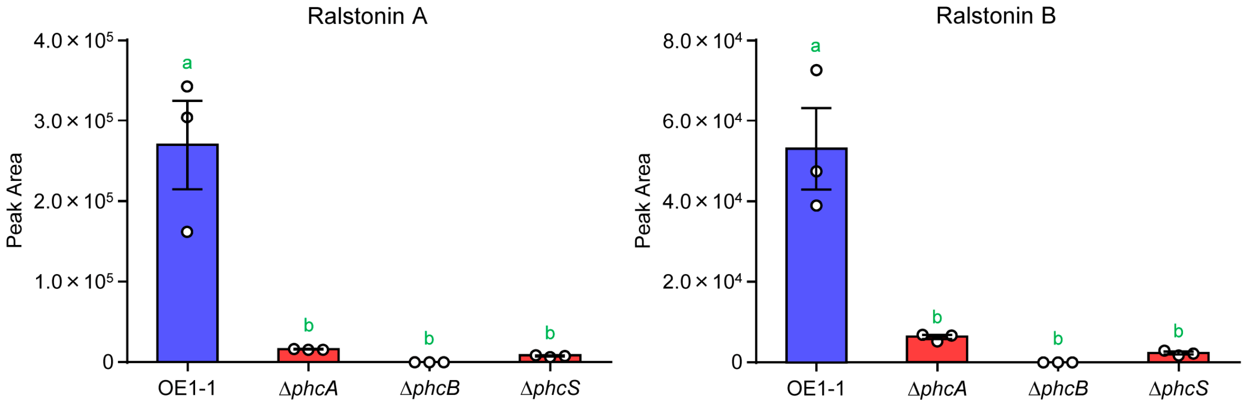

Figure S1. Comparing ralstonin production in OE1-1, Δ*phcA*, Δ*phcB*, and Δ*phcS* strains. The graph on the left shows ralstonin A data and on the right, ralstonin B data. Compounds from different strain culture extracts were analyzed by LC/MS. Error bars indicate the mean ± SEM (n = 3). Green letters above error bars indicate significant differences ($p < 0.05$, Tukey's test).

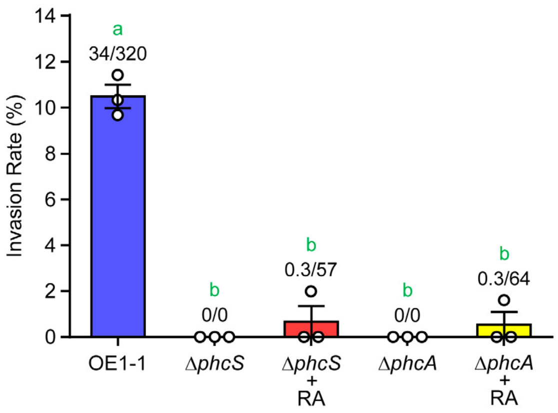

Figure S2. Invasion rates of Δ*phcS* and Δ*phcA* and their response to ralstonin A. Ralstonin A (RA) was used at 0.1 nmol/disk. OE1-1 is a positive control. The error bars show the mean ± SEM (*n* = 3). The numbers above the bars indicate infected spores/total spores. The green letters above the bars show significant differences ($p < 0.05$, Tukey's test).

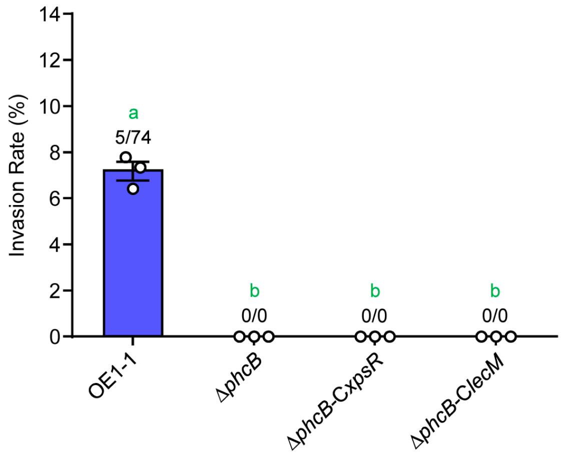

Figure S3. Invasion rates of Δ*phcB*, Δ*phcB*-C*xpsR*, and Δ*phcB*-C*lecM* into *F. oxysporum* chlamydospores. The error bars show the mean ± SEM (n = 3). The numbers above the bars show infected spores/total spores. The green letters above the bars show significant differences ($p < 0.05$, Tukey's test).

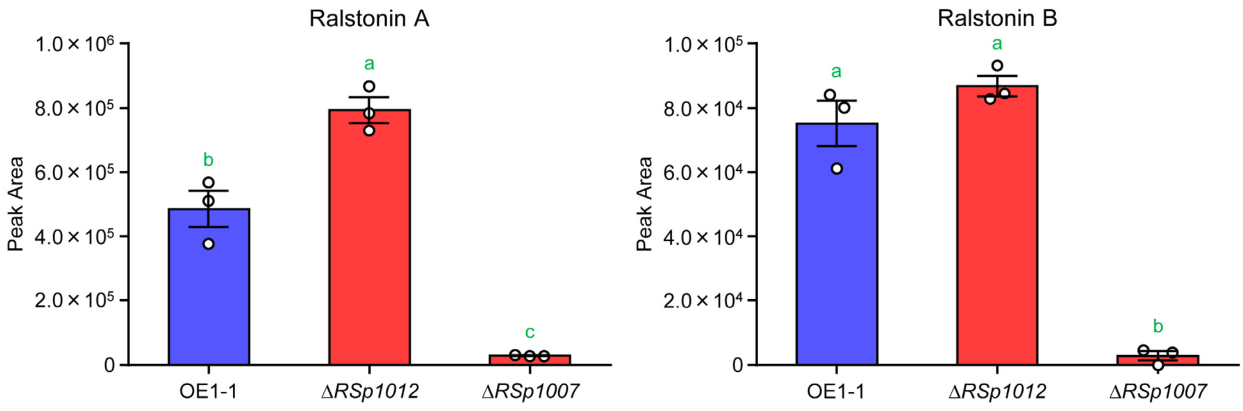

Figure S4. Comparison of ralstonin production in OE1-1, Δ*RSp1012*, and Δ*RSp1007*. On the left are the data for ralstonin A and on the right are the data for ralstonin B. Compounds from different strain culture extracts were analyzed by LC/MS. The error bars show the mean ± SEM (n = 3). The green letters above the bars show significant differences ($p < 0.05$, Tukey's test).

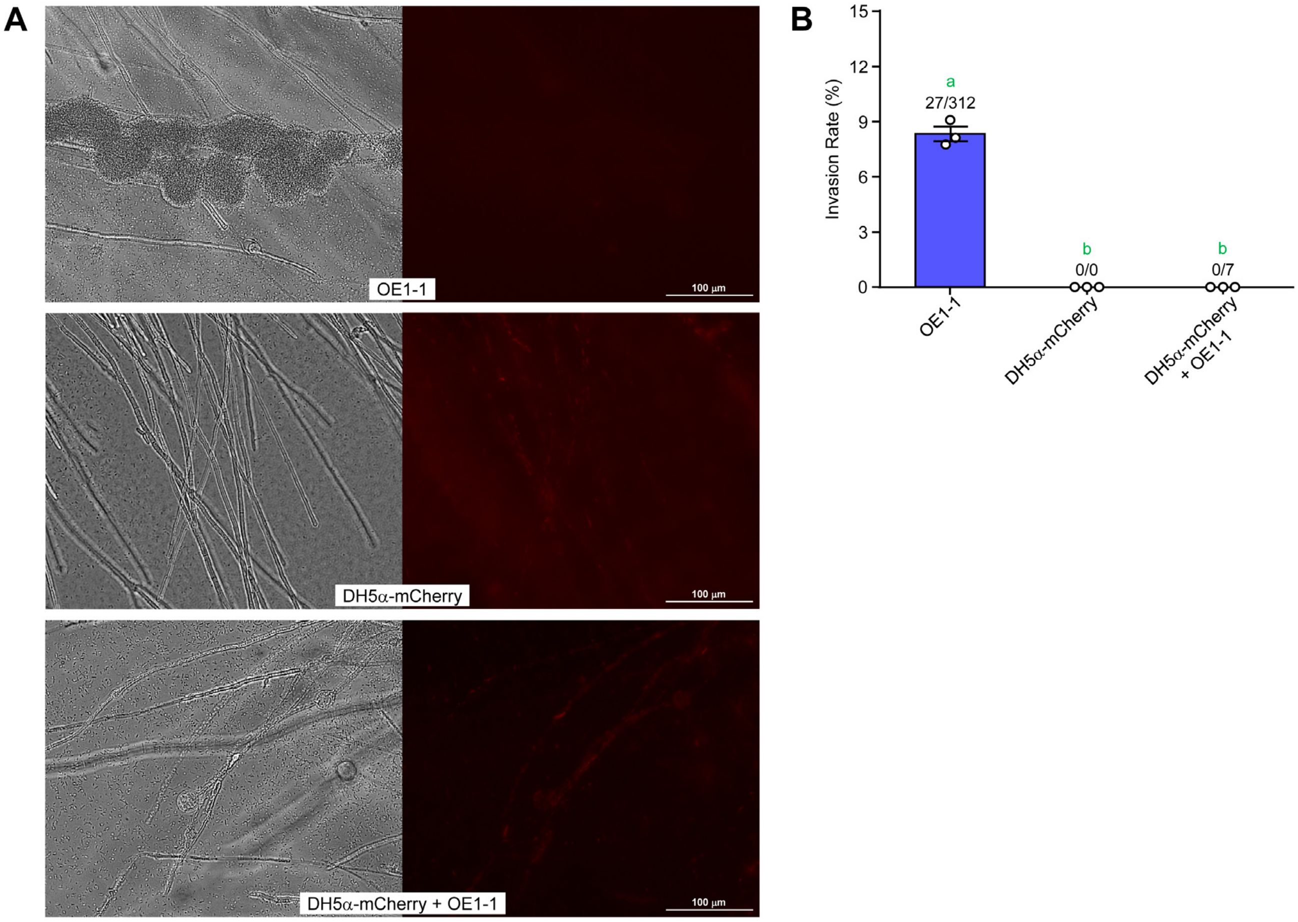

Figure S5. Evaluation of *E. coli* DH5α-mCherry biofilm formation and invasion ability (A) The photos of coculturing of *F. oxysporum* with OE1-1, *E. coli* DH5α-mCherry, and both. The bright field image is on the left, and the fluorescence image is on the right. (B) Invasion rates of OE1-1 and *E. coli* DH5α-mCherry into *F. oxysporum* chlamydospores. *E. coli* DH5α-mCherry was not parasitized even when mixed with OE1-1 and the mixture was used. The error bars show the mean ± SEM (n = 3). The numbers above the bars indicate infected spores/total spores. The green letters above the bars show significant differences ($p < 0.05$, Tukey's test).

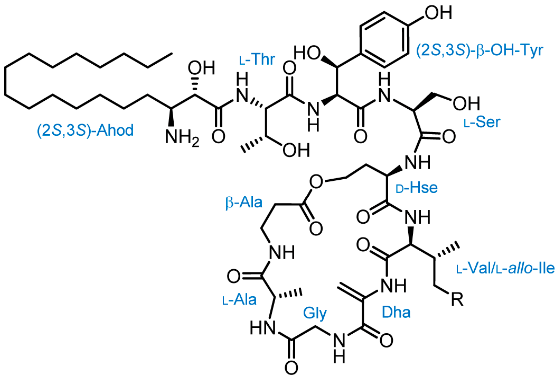

Ralstopeptin A: R = H
Ralstopeptin B: R = CH_3

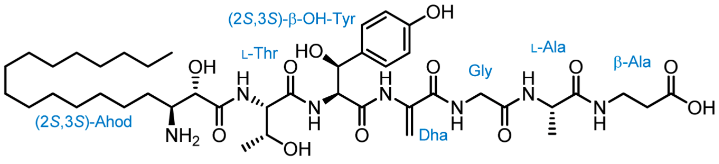

Ralstoamide A

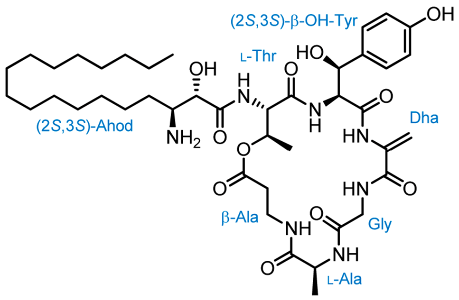

Ralstoamide B

Figure S6. Ralstopeptin A/B and ralstoamide A/B structures.

Table S1. Plasmids and bacterial strains used in this study

Plasmids/Bacterial strains	Characteristics	Source
**Plasmids**		
pDSK519	Km^r RSF1010 derivative, Km^r *mob*$^+$, *lacZ*	Keen *et al.*, 1988
pDSK-GFP	Km^r pDSK519 with *psbA*-RBS-GFP	This study
pDSK-xpsR	Km^r pDSK519 with *psbA*-RBS-*xpsR*	This study
pDSK-lecM	Km^r pDSK519 with *psbA*-RBS-*lecM*	This study
pDSK-mCherry	Km^r pDSK519 with *psbA*-RBS-mCherry	This study
pK18mobsacB	Km^r, *oriT*(RP4), *sacB*, *lacZ*α	Kvitko and Collmer, 2011
pΔRSp0275	pK18mobsacB derivative carrying a 900-bp DNA fragment for *RSp0275* deletion, Km^r	This study
pΔRSp0924	pK18mobsacB derivative carrying a 900-bp DNA fragment for *RSp0924* deletion, Km^r	This study
pΔRSc0818	pK18mobsacB derivative carrying a 900-bp DNA fragment for *RSc0818* deletion, Km^r	This study
pΔRSp0138	pK18mobsacB derivative carrying a 900-bp DNA fragment for *RSp0138* deletion, Km^r	This study
pΔRSp0161	pK18mobsacB derivative carrying a 900-bp DNA fragment for *RSp0161* deletion, Km^r	This study
pΔgspD	pK18mobsacB derivative carrying a 900-bp DNA fragment for *gspD* deletion, Km^r	This study
pΔRSp1012	pK18mobsacB derivative carrying a 900-bp DNA fragment for *RSp1012* deletion, Km^r	This study
pΔRSp1007	pK18mobsacB derivative carrying a 900-bp DNA fragment for *RSp1007* deletion, Km^r	This study
***Escherichia coli* strain**		
DH5α	F^-, φ 80d*lacZ* ΔM15, Δ(*lacZYA-argF*)U169, *deoR*, *recA*1, *endA*1, *hsdR*17(rK^-, mK^+), *phoA*, *supE*44, λ^-, *thi*-1, *gyrA*96, *relA*1	Takara Bio
DH5α-mCherry	DH5α harboring pDSK-mCherry, Km^r, mCherry	This study
**RSSC strains**		
OE1-1	Wild-type strain, phylotype I, race 1, biovar 4	Kanda et al., 2003
OE1-1-*gfp*	OE1-1 harboring pDSK-GFP, Km^r, GFP	This study
Δ*rmyA*	*rmyA*-deletion mutant of OE1-1	24
Δ*rmyA-gfp*	Δ*rmyA* harboring pDSK-GFP, Km^r, GFP	This study
Δ*phcB*	*phcB*-deletion mutant of OE1-1	21
Δ*phcB-gfp*	Δ*phcB* harboring pDSK-GFP, Km^r, GFP	This study
Δ*phcS*	*phcS*-deletion mutant of OE1-1	21
Δ*phcS-gfp*	Δ*phcS* harboring pDSK-GFP, Km^r, GFP	This study
Δ*phcA*	*phcA*-deletion mutant of OE1-1	21
Δ*phcA-gfp*	Δ*phcA* harboring pDSK-GFP, Km^r, GFP	This study
Δ*RSp0275*	*RSp0275*-deletion mutant of OE1-1	This study
Δ*RSp0275-gfp*	Δ*RSp0275* harboring pDSK-GFP, Km^r, GFP	This study

Δ*RSp0924*	*RSp0924*-deletion mutant of OE1-1	This study
Δ*RSp0924-gfp*	Δ*RSp0924* harboring pDSK-GFP, Km^r, GFP	This study
Δ*egl*	*egl*-deletion mutant of OE1-1	21
Δ*egl-gfp*	Δ*egl* harboring pDSK-GFP, Km^r, GFP	This study
Δ*cbhA*	*cbhA*-deletion mutant of OE1-1	Senuma et al., 2023
Δ*cbhA-gfp*	Δ*cbhA* harboring pDSK-GFP, Km^r, GFP	This study
Δ*RSc0818*	*RSc0818*-deletion mutant of OE1-1	This study
Δ*RSc0818-gfp*	Δ*RSc0818* harboring pDSK-GFP, Km^r, GFP	This study
Δ*RSp0138*	*RSp0138*-deletion mutant of OE1-1	This study
Δ*RSp0138-gfp*	Δ*RSp0138* harboring pDSK-GFP, Km^r, GFP	This study
Δ*RSp0161*	*RSp0161*-deletion mutant of OE1-1	This study
Δ*RSp0161-gfp*	Δ*RSp0161* harboring pDSK-GFP, Km^r, GFP	This study
Δ*gspD*	*gspD*-deletion mutant of OE1-1	This study
Δ*gspD-gfp*	Δ*gspD* harboring pDSK-GFP, Km^r, GFP	This study
Δ*hrpB*	*hrpB*-deletion mutant of OE1-1	Yoshimochi et al., 2009
Δ*hrpB-gfp*	Δ*hrpB* harboring pDSK-GFP, Km^r, GFP	This study
Δ*hrpG*	*hrpG*-deletion mutant of OE1-1	Yoshimochi et al., 2009
Δ*hrpG-gfp*	Δ*hrpG* harboring pDSK-GFP, Km^r, GFP	This study
Δ*epsB*	*epsB*-deletion mutant of OE1-1	43
Δ*epsB-gfp*	Δ*epsB* harboring pDSK-GFP, Km^r, GFP	This study
Δ*epsB*-mCherry	Δ*epsB* harboring pDSK-mCherry, Km^r, mCherry	This study
Δ*lecM*	*lecM*-deletion mutant of OE1-1	42
Δ*lecM-gfp*	Δ*lecM* harboring pDSK-GFP, Km^r, GFP	This study
Δ*RSp1012*	*RSp1012*-deletion mutant of OE1-1	This study
Δ*RSp1012-gfp*	Δ*RSp1012* harboring pDSK-GFP, Km^r, GFP	This study
Δ*RSp1007*	*RSp1007*-deletion mutant of OE1-1	This study
Δ*RSp1007-gfp*	Δ*RSp1007* harboring pDSK-GFP, Km^r, GFP	This study
epsB-comp	A transformant of Δ*epsB* with native *chpA*, Gm^r	43
epsB-comp-*gfp*	*epsB*-comp harboring pDSK-GFP, Km^r, GFP	This study
lecM-comp	A transformant of Δ*lecM* with native *lecM*, Gm^r	42
lecM-comp-*gfp*	*lecM*-comp harboring pDSK-GFP, Km^r, GFP	This study
Δ*phcB*-C*xpsR*	Δ*phcB* harboring pDSK-xpsR, Km^r, XpsR	This study
Δ*phcB*-C*xpsR-gfp*	Δ*phcB* harboring pDSK-xpsR-GFP, Km^r, XpsR, GFP	This study
Δ*phcB*-C*lecM*	Δ*phcB* harboring pDSK-lecM, Km^r, LecM	This study
Δ*phcB*-C*lecM-gfp*	Δ*phcB* harboring pDSK-lecM-GFP, Km^r, LecM, GFP	This study
